# Lacking P2X7-receptors protects substantia nigra dopaminergic neurons and hippocampal-related cognitive performance from the deleterious effects of high-fat diet exposure in adult male mice

**DOI:** 10.3389/fnut.2024.1289750

**Published:** 2024-01-25

**Authors:** Chiara Rossi, Mariarosaria Distaso, Francesco Raggi, Claudia Kusmic, Francesco Faita, Anna Solini

**Affiliations:** ^1^Department of Surgical, Medical, Molecular and Critical Area Pathology University of Pisa, Pisa, Italy; ^2^National Research Council, Pisa, Italy

**Keywords:** brain, high fat diet, insulin, parkin, P2X7 receptor

## Abstract

**Background:**

Dietary fat consumption, involved in the pathogenesis of insulin resistance and impaired glucose metabolism, is linked with decline in cognitive functions, dementia, and development of Parkinson’s disease and Alzheimer’s disease. Mature IL-1β, requiring the activation of the P2X7 receptor (P2X7R)-inflammasome complex, is an important mediator of neuroinflammation. The aim of the study was to test whether P2X7R activation might interfere with systemic and cerebral metabolic homeostasis.

**Methods:**

We treated WT and P2X7R KO mice with a high-fat diet (HFD) for 16 weeks, evaluating the effects on the Substantia Nigra and Hippocampus, target areas of damage in several forms of cognitive impairment.

**Results:**

HFD-treated WT and P2X7R KO mice showed a different brain mRNA profile of Insulin and Igf-1, with these genes and relative receptors, more expressed in KO mice. Unlike P2X7R KO mice, WT mice treated with HFD displayed a diameter reduction in dopaminergic neurons in the Substantia Nigra, accompanied by an increased IBA1 expression in this area; they also showed poor performances during Y-Maze and Morris Water Maze, tasks involving Hippocampus activity. Conversely, Parkin, whose reduction might promote neuronal cell death, was increased in the brain of P2X7R KO animals.

**Conclusion:**

We report for the first time that HFD induces damage in dopaminergic neurons of the Substantia Nigra and a Hippocampus-related worse cognitive performance, both attenuated in the absence of P2X7R. The involved mechanisms might differ in the two brain areas, with a predominant role of inflammation in the Substantia Nigra and a metabolic derangement in the Hippocampus.

## Introduction

In the last decade, several epidemiological studies have linked hypercholesterolemia and high-fat diet consumption to cognitive function decline and dementias (1–3). Metabolic alterations related to a high-fat consumption generally include impaired glucose metabolism and insulin resistance, commonly observed during neurodegenerative chronic diseases such as Parkinson’s disease (PD) (4) and Alzheimer’s disease (AD) (5). A longitudinal population study performed in South Korea showed as incidence, and the risk of PD increased gradually with the number of metabolic syndrome components, suggesting that neurodegenerative disease and metabolic abnormalities could share pathophysiological mechanisms (6); similarly, non-pharmacologic interventions can improve insulin sensitivity and ameliorate cognitive functions in AD individuals (7). Neuroimaging studies have shown protective effects of the Mediterranean Diet on neuronal structures, and early morphological changes are linked to neurodegeneration and AD (8).

Although the early stages of AD and PD are characterized by a dysfunction in specific neuronal clusters (substantia nigra in PD, hippocampal and cortical region in AD), these diseases share some pathophysiological mechanisms, such as oxidative stress, mitochondrial dysfunction, glutamate toxicity, and neuroinflammation, that largely contribute to neuronal death (9, 10); in other words, metabolic alterations in different neuronal cells could be recognized as a common link of different neurodegenerative diseases.

An extensive literature, based on animal models, supports the link between diet, metabolic alterations, and cognitive impairment. Rats developing overweight and insulin resistance after a diet treatment show poorer cognitive performances than control animals in spatial learning ability task, a reduced hippocampal plasticity (11–13) and increased neuronal apoptosis in the hippocampus and hypothalamus (14, 15). Mice fed with high-fat/high-cholesterol diet show impaired working memory, associated with a hippocampus dysregulation, coupled with increased brain mRNA expression of pro-inflammatory cytokines/mediators (16). In another experimental setting, high-fat diet-treated mice display an impaired cognition, as measured by the Stone T-maze, accompanied by brain inflammation and brain-derived neurotrophic factor (BDNF) reduction. Interestingly, such alterations can be induced only by very high-fat (60%) dietary regimens (17).

Inflammation plays a prominent role in the pathogenesis and progression of several neurodegenerative diseases, and interleukin-1β (IL-1β) is the main inducer of neuroinflammation. Pro-inflammatory cytokines can either cross the blood–brain barrier or it can be produced by resident cells (microglia, astrocytes, and endothelial cells). In a rat model of PD, IL-1β is abundantly represented in the microglia surrounded by Lewy bodies, where it mediates inflammation and consequent dopaminergic neuron degeneration in the central nervous system (CNS) (18). Maturation and release of IL-1β require the activation of the P2X7 receptor (P2X7R)-inflammasome complex. The direct role of P2X7R in neuroinflammation has been extensively examined (19), and it is mainly related to microglia and astrocyte (20) activation. However, P2X7R activation also involves various metabolic pathways (21), such as excitotoxicity induced by glutamate release from neurons and astrocytes and neuroinflammation produced by IL-1β and other cytokine release (22). Hence, P2X7R can enhance the production of reactive oxygen species (ROS) that aggravate protein misfolding and neuronal damage, induce direct or indirect cell death, downregulate BDNF and impair neuroplasticity.

Therefore, we hypothesize that P2X7R activation might interfere with systemic and cerebral metabolic homeostasis of dopaminergic neurons with special regard to the connection between insulin signaling and low-grade inflammation.

## Materials and methods

### Animals

The study protocol was approved by the Italian Minister for Animal Care (protocol number #943/2015-PR, approved November 2016). Twelve wild type (WT) (strain C57BL6J, ENVIGO, Udine, Italy) and twelve P2X7R KO male mice (C57Bl6 background [*P2rx7^tm1Gab^*/J]), originally generated from Pfizer and purchased from Jackson Laboratory, through Charles River, Lecco, Italy) were housed in a pathogen-free stabularium in accordance with the principles of Italian Minister for Animal Care (protocol authorization number #943/2015-PR) and maintained under controlled ambient illumination on a 12 h light/dark cycle and free access to food. Each mouse strain was divided into two groups (six animals each) and treated for 16 weeks with a normal fat diet (NFD, a routinely diet with 4.0% fats of total calories (Teklad Global Rodent Diet 2016, Envigo, Udine, Italy) and a high fat diet (HFD, 60% fats of total calories, PF4215, Research Diets Mucedola, Settimo Milanese, Italy). We selected such commercial HFD with controlled fat amount and composition, rather than preparing a home-made diet, to avoid inaccuracy and potential alterations of the diet energetic balance over time. A further group of five WT animals fed with HFD undergone an intraperitoneal injection of a P2X7R antagonist (Tocris, AZ10606120 100 nM, 100 μL three times/week) over the whole period of dietary treatment, to evaluate the effect of the receptor antagonist on glucose tolerance. Animals started the dietary treatments at the age of 10 weeks.

### Biochemical parameters

During the study, *ad libitum* fed mice were weighed every week; plasma glucose, triglycerides, cholesterol, and aspartate aminotransferase (AST) were measured at the baseline and at week 16. Metabolic measures were performed by a digital multifunction meter (MultiCare-in, BSI, Milan, Italy); AST activity was measured by an enzymatic method in a Synchron Clinical System (Beckman Coulter, Fullerton, CA, United States).

At the end of the 16 weeks of NFD or HFD, an intraperitoneal glucose tolerance test (IPGTT) was performed. In brief, after 6 h of fasting, mice received a bolus of 0.05 g glucose in the intraperitoneal cavity, and plasma glucose was measured at basal, 30 min, 60 min, and 120 min using the above-reported multimeter.

### Behavioral test

At the end of the dietary treatments, P2X7R KO and WT mice carried out two behavioral tests (Y-Maze and Morris Water Maze), allowing them to recover for 2 days between the two tests. Each mouse underwent such tests starting from 9.00 am in a behavioral test room. Y-Maze is a test measuring spatial working memory; it is executed in a compartment made of three arms arranged in Y-shape, testing rodent preference to explore new ambient to find an escape (over the course of multiple arm entries, mice should show a tendency to enter a less recently visited arm). Each mouse was placed in the center of the maze, then the number of arm entries and the number of triads were recorded by a video tracking system for 8 min; an entry occurred when all four limbs were within the arm. Video Tracking Software (Panlab, Barcelona, Spain) calculates the percentage of correct alternation, the mean speed, and the travelled total distance.

The typical equipment of Morris Water Maze consists of a circular pool filled with opaque water at approximately 21°C. The water tank was surrounded by three visual cues, which are useful to create a spatial map (the ambient around the pool was made neutral covering the objects present). A platform was placed approximately 1.5 cm below the water surface, remaining in a fixed location relative to the cues during the trial. The procedure consisted of 5 days of trial, four training per day. For each trial, the animal was placed into the water at different start positions, allowing it to swim for 60 s; if the mouse did not find the platform in this time, it was placed manually on the platform; in any case, it stayed there for 20 s before returning to its cage. Mice were monitored by a video tracking system placed directly above the water tank, measuring Escape Latency (EL) and Time in Quadrant through a Video Tracking Software (Panlab).

### Neuroanatomy

After the behavioral tests, mice were deeply anesthetized with sevoflurane; the brains were removed from the skull and sagittally cut. Half part was immediately frozen on dry ice for RNA isolation; the other hemisphere was fixed by immersion in 10% Neutral Buffered Formalin for 24 h at 4°C; after the washing step in phosphate buffer (PBS), brains were left overnight at 4°C in a cryoprotection solution containing 30% sucrose in PBS; the next day, they were frozen in optimal cutting temperature (OCT) compound (Histo-Line Laboratories, Milan, Italy).

Coronal brain sections cut in the cryostat and mounted on non-charged slides were immediately observed with a microscope, and sections containing the midbrain/substantia nigra (SN) or hippocampus (HIP) were established on the base of mouse brain atlas. In the case of half brain frozen, the specific area was dissected out from each section using a needle, and the tissue was used for the subsequent RNA extraction; in the case of half brain fixed, the sections containing SN or HIP were recovered in PBS for free-floating immunostaining.

### Gene expression

RNA extraction was performed with QIAcube (QIAGEN, Hilden, Germany), a robotic workstation for automated purification of nucleic acids, using RNeasy mini kit (QIAGEN), following the manufacturing protocol. The starting material (6×50 μm brain sections for each animal) was lysed using the TissueLyser (QIAGEN). After a spectrophotometric quantification by NanoDrop 2000c (Thermo Fisher Scientific, Waltham, MA, United States), 250 ng of total RNA from brain sections were reverse transcripted (RT) to single-stranded cDNA by High-Capacity cDNA Reverse Transcription Kit (Thermo Fisher Scientific). Gene expression analysis was executed in Eco real-time instrument (Illumina Inc., San Diego, CA, United States) using following TaqMan Assays (Thermo Fisher Scientific): Nrlp3 (Mm00840904_m1), Parkin (Mm01323528_m1), α-synuclein (Mm01188700_m1), Gapdh (Mm99999915_g1), Insulin (Mm00731595_g1), Insulin rec (Mm01211875_m1), Igf1 rec (Mm00439560_m1), Igf1r (Mm00802831_m1), Gsk-3β (Mm00444911_m1), and Foxo3 (Mm01185722_m1). Amplifications were normalized by housekeeping gene, and quantitation of gene expression was performed using the ∆∆C_t_ calculation, where C_t_ is the threshold cycle. The amount of the target gene, normalized to GAPDH and relative to WT animal, is given as 2^−∆∆Ct^.

### Western blot

Total proteins were extracted from 15 sections of 50 μm, to measure total and phosphorylated ERK 1/2, JNK, AKT, GSK-3β, and AMPK by Western Blot (WB) analysis. Each sample was homogenized with 200 μL of lysis Buffer (based on NP40 mix, Invitrogen) and modified to protect the protein phosphate groups. After a centrifugation step at 14,000 rpm at 4°C to precipitate cellular debris, the protein content was evaluated by a colorimetric assay (Quick Start Bradford Protein Assay, Bio-Rad Laboratories, Hercules, CA, United States). For the WB experiments, 30 μg of total proteins was diluted in SDS loading buffer and heated at 100°C for 5 min. Samples were separated on precast gels (Any kD Mini-Protean TGX gels, Bio-Rad), run at constant voltage in a vertical electrophoresis apparatus, and subsequently transferred to a polyvinyl difluoride (PVDF) membrane (Millipore-Merck KGaA, Darmstadt, Germany) with a semi-dry blotting process (TransBlot Turbo, Bio-Rad Laboratories). After a treatment with Blocking Solution (1 h at room temperature) to saturate aspecific binding sites, blots were incubated overnight at 4°C with diluted 1:1000 primary antibodies: AKT 9272; pAKT 9271;GSK-3β 12456; pGSK-3β 5558; AMPK 2532; and p-AMPK 2331 (all from Cell Signaling Technology, Danvers, MA, United States). The next day, identification of specific bands was performed by a chemiluminescence reaction: after 1 h of incubation with secondary antibody conjugated with horseradish peroxidase (HRP); the membrane was covered with a solution containing the chemiluminescent substrate (Clarity Western ECL, Bio-Rad Laboratories); in the presence of H_2_O_2_, HRP catalyzes the oxidation of luminol, which then generates light only where the target protein is present. The lighted bands, detected by a digital camera (Versadoc instrument, Bio-Rad Laboratories), were quantified by evaluating their optical density by ImageJ (Software) and normalized with GAPDH bands (mab374, Millipore).

### Immunofluorescence

Coronal sections (50 μm thick) of SN or HIP were cut on a cryostat and collected in PBS; after a post-fixation step (1 h at room temperature) in paraformaldehyde 4% and three washes with PBS, serial sections were treated for 1–2 h at room temperature with a blocking solution, to saturate aspecific binding sites. Samples were incubated overnight at 4°C with specific primary antibodies: P2X7R (APR-004 Alomone Labs, Jerusalem, Israel); NRLP3 (AG-20B-0014 Adipogen Life Sciences, San Diego, CA, United States); GFAP (ab7260 Abcam, Cambridge, UK); TH (ab75875 Abcam); and IBA1 (sc-32725 Santa Cruz Biotechnology, Dallas, TX, United States). The following day, immunoreactivity was revealed using secondary antibodies against mouse or rabbit antigen (Alexa Fluor 594-goat@rabbit and 488-goat@mouse antibodies, Invitrogen, Thermo Fisher Scientific), depending on the primary antibody host species.

The images used for the quantitative analysis of immunofluorescence were acquired with a TCS SP8 confocal microscope (Leica Microsystems, Wetzlar, Germany). After a preliminary analysis of different samples to establish the best conditions for the instrument, the confocal setting was held constant within all experimental sessions. All image analyses were performed using ImageJ (free image processing program released from National Institute of Health, United States).

In mouse brains, similar stacks of optical sections were acquired for each animal.

### Immunoreactivity quantification

Threshold area was established on the basis of average background signal. In brief, for each image transformed in grayscale, the grey value in three different non-signal areas was measured using the mean value to set the threshold; in the binary image obtained, the percentage area of positive pixel was calculated. In the case of not specific green signal in vessels, these parts were masked before calculating the threshold area.

### Cell diameter

Diameter of TH-positives cells was measured in stack image projection by drawing a line through the center of cell body with the specific tool of ImageJ software. The length of the line, in pixels, was converted into μm according to the calibration bar shown in the picture.

### Statistical analysis

The results are expressed as mean ± standard deviation (SD). Statistical analysis was carried out using analysis of variance with post-hoc Bonferroni correction, Mann–Whitney test for non-parametric data, and Student’s unpaired *t*-test for comparison between groups. Data were deemed significant when *p* < 0.05.

## Results

### Metabolic effects of HFD on WT and KO animals

HFD induced a significant weight gain in all animals (Supplementary Figure S1), while it slightly affected their biochemical parameters: only cholesterol at the end of the treatment was increased in both WT and P2X7R KO mice, while glucose showed a raising trend in HFD-treated animals (Supplementary Table S1).

To better detect a potential impairment in glucose metabolism, at the end of the treatment, WT and P2X7R KO mice underwent an IPGTT. As expected, animals receiving HFD showed a greater Area Under Curve (AUC, WH > WN and KH > KN, *p* < 0.01; Supplementary Figure S2); however, the increment of such area was similar in WT and P2X7R KO mice, suggesting that P2X7R does not influence the glucose metabolism in our experimental model. Indeed, the intraperitoneal injection of the P2X7R receptor antagonist did not modify such trend (Supplementary Figure S2). WT HFD-treated mice with or without antagonist displayed the same AUC.

### Insulin signaling

**Figure 1:**
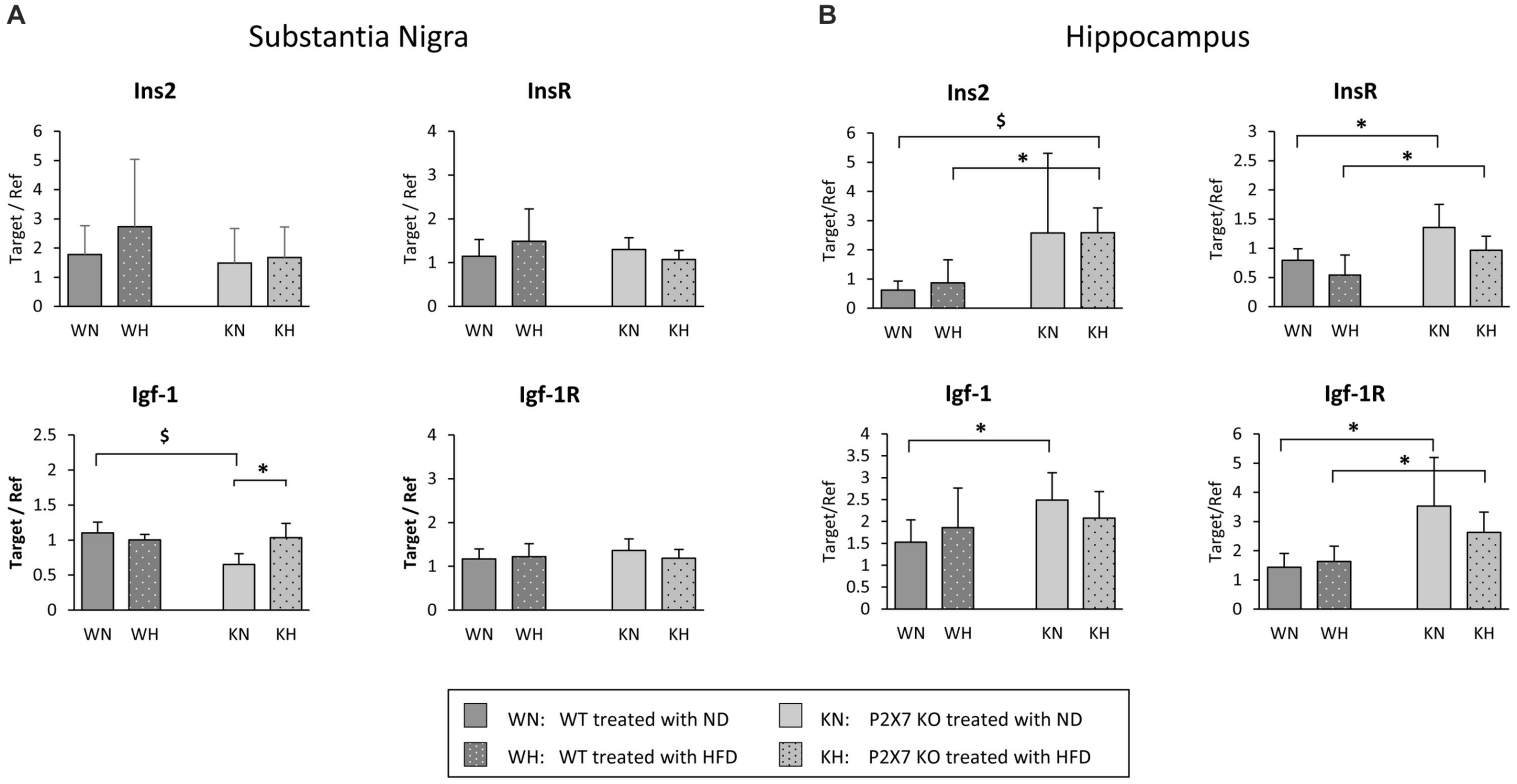
mRNA quantification of Insulin, IGF-1, and relative receptors in the Substantia Nigra **(A)** and the Hippocampus **(B)** of WT and P2X7R KO mice fed with normal (ND) or high-fat diet (HFD). Data are mean ± SD (*n* = 6 for each group). *p < 0.05 ^$^*p* < 0.01.

**Figure 2:**
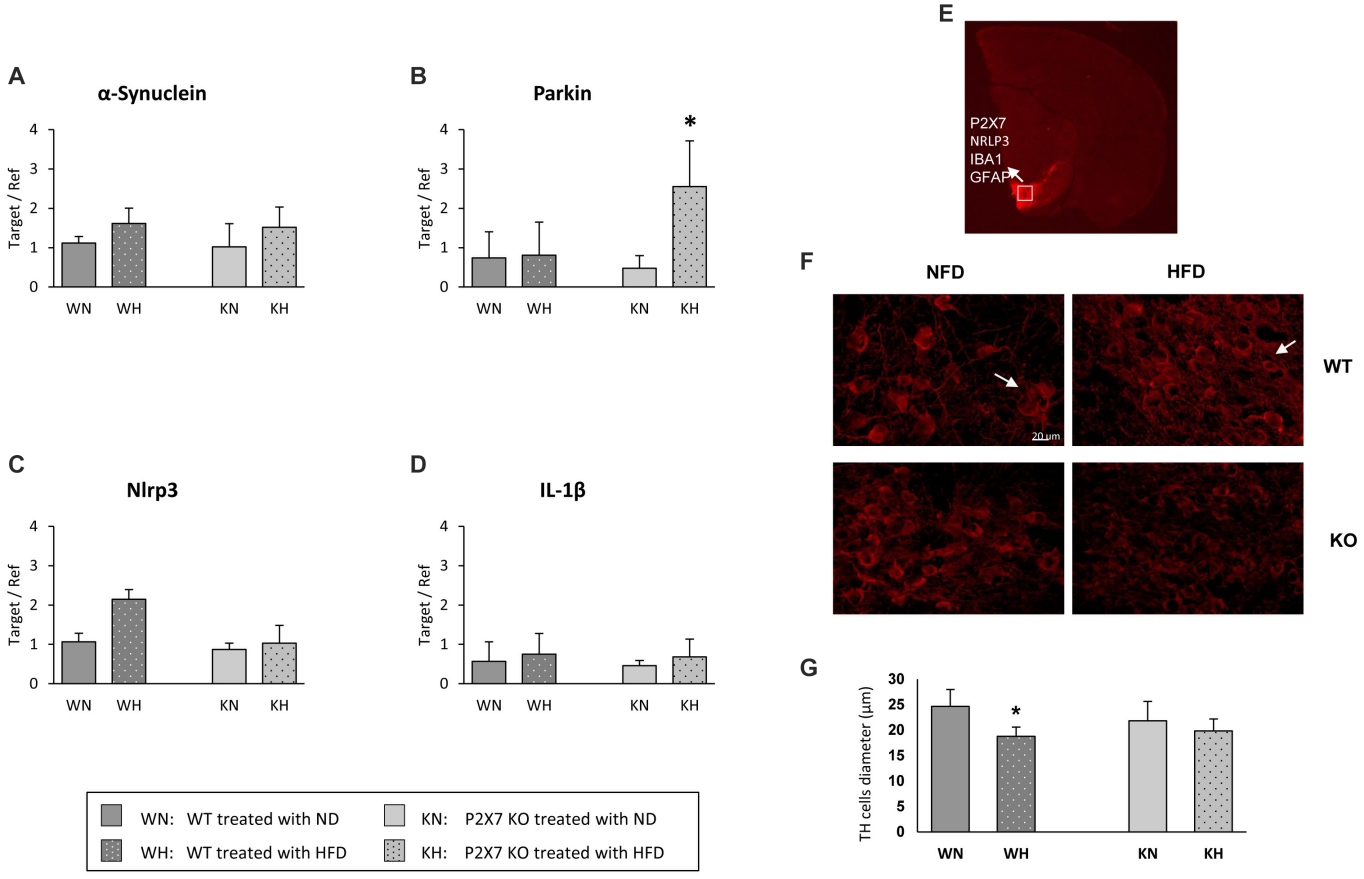
α-synuclein **(A)**, Parkin **(B)**, NRLP3 **(C)**, and IL-1β **(D)** mRNA quantification in the Substantia Nigra of WT and P2X7R KO mice fed with normal (ND) or high-fat diet (HFD). Data are mean ± SD (*n* = 6 for each group). *p < 0.001 ND vs. HFD. Representative murine half brain where SN is highlighted by TH-immunostaining (red); the white square indicates the acquisition area for all antibodies used in this study **(E)**. Representative images of tyrosine hydroxylase (TH)-positive cells in the Substantia Nigra of WT and P2X7 KO mice **(F)**. Relative quantification of cell diameter in the whole study group **(G)**. Data are mean ± SD (*n* = 6 for each group). *p < 0.001 vs. WN.

Expression of insulin and IGF-1 genes, as well as of their respective receptors, were analyzed in SN and HIP. Data are shown in Figure 1. They resulted mainly altered in HIP, where they were more expressed in KO when compared with WT mice (Figure 1B). In SN, differences between WT and P2X7R KO animals were not evident: the expression of insulin, insulin receptor, and IGF-1 receptor was similar in all groups (Figure 1A). IGF-1 seemed to be influenced by HFD only in KO animals (KN vs. KH, *p* = 0.021; Figure 2A); of interest, NFD determined a lower IGF-1 expression in KO mice (WN vs. KN, *p* = 0.006).

Due to this different brain pattern of expression, we decided to perform separately the analysis of the two areas (HIP and SN).

### Substantia Nigra

The SN is an area characterized by a massive loss of dopaminergic neurons in PD; α-synuclein expression levels resulted tendentially similar in the two mouse strains, with an upward trend in both HFD experimental groups (Figure 2A). Conversely, Parkin showed an intriguing expression profile: its expression did not vary in WT animals treated with HFD, while it was strongly increased in P2X7R KO mice (Figure 2B). Of note, the NLRP3 inflammasome followed exactly an opposite trend, being upregulated in the SN of WT animals treated with HFD, not varying in KO mice (Figure 2C); this trend was lost in the final product of inflammasome activation, IL-1β: its expression was not influenced by diet in any experimental group (Figure 2D).

To confirm SN as a target of the specific inflammatory damage induced by HFD, we also looked at tyrosine hydroxylase (TH)-positive neurons, double-stained with the following antibodies: P2X7R, NRLP3, Iba1, and GFAP. In Figure 2E, the white square indicates the target area of confocal acquisition. In WT animals treated with HFD, these dopaminergic cells showed a significantly reduced diameter (Figure 2F), accompanied by a less intense immunoreactivity signal with respect to NFD-treated animals (Figure 2G).

**Figure 3:**
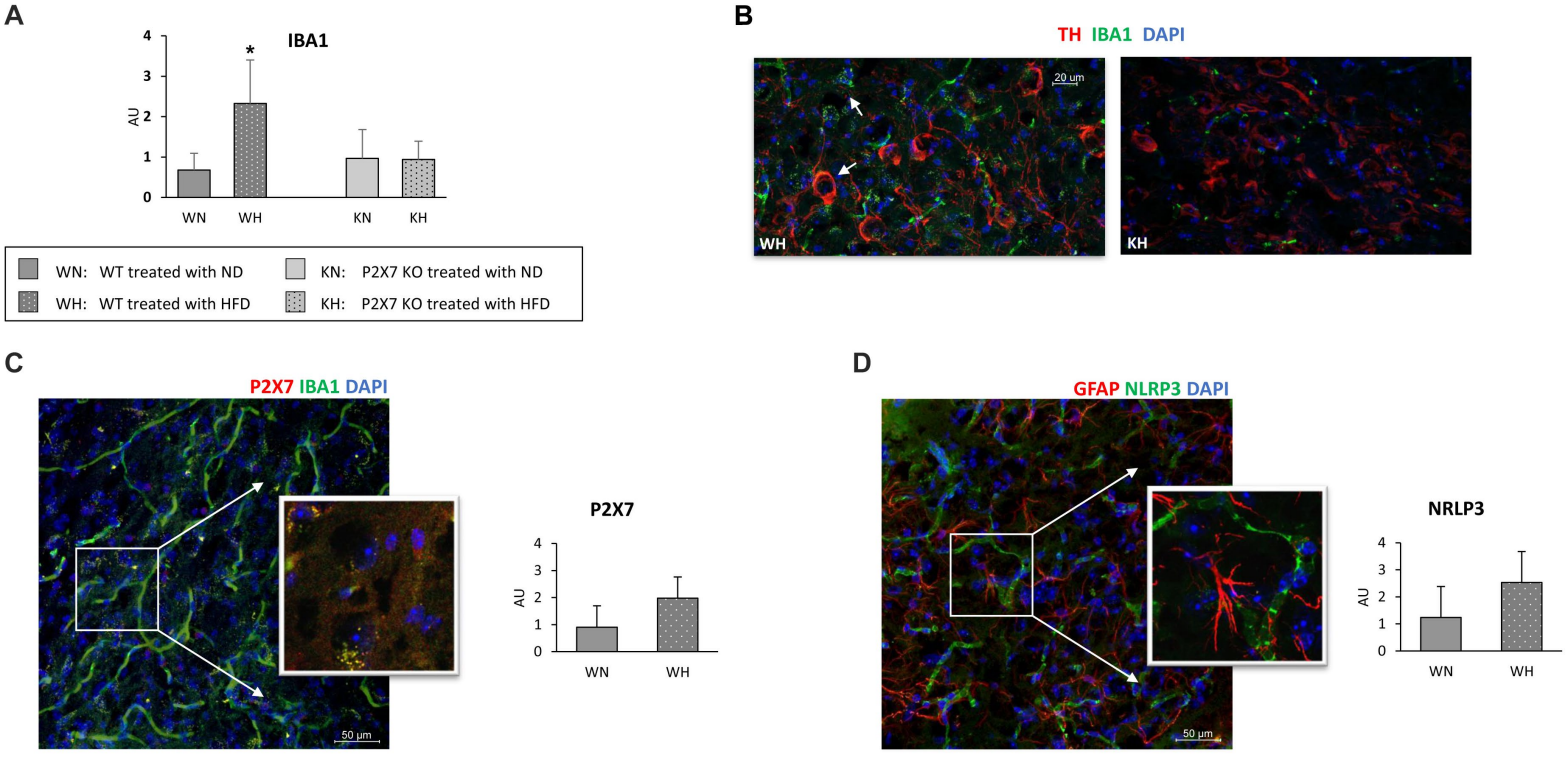
Activated microglia labeled with Iba1 in SN of WT and KO mouse brain treated with normal (ND) or high-fat diet (HFD); quantification in the whole study group **(A)**, and representative confocal image in WT and KO mice treated with HFD **(B)**. Representative immunofluorescence images acquired in the Substantia Nigra of WT mice (inserts are single focal planes of indicated area): on the left, P2X7R colocalizes with Iba1 **(C)**; on the right GFAP and NRLP3 do not colocalize **(D)**. Graphs report the quantification of immunoreactivity. Data are mean ± SD (*n* = 6 for each group). *p < 0.001 vs. WN.

Figure 3A shows the quantification of activated microglia (IBA1-positive cells): it was mostly expressed in WT animals treated with HFD (a representative confocal image of WT and P2X7R KO mice treated with HFD is presented in Figure 3B): in P2X7R KO mice, HFD did not induce any difference. Furthermore, in the same area, WT mice fed with HFD displayed a trending higher P2X7R (Figure 3C) and NLRP3 (Figure 3D) protein expression. Of note, P2X7R colocalized with microglia (identified by Iba1-positive cells), and NLRP3 did not colocalize with astrocytes (identified by GFAP antibody).

**Figure 4:**
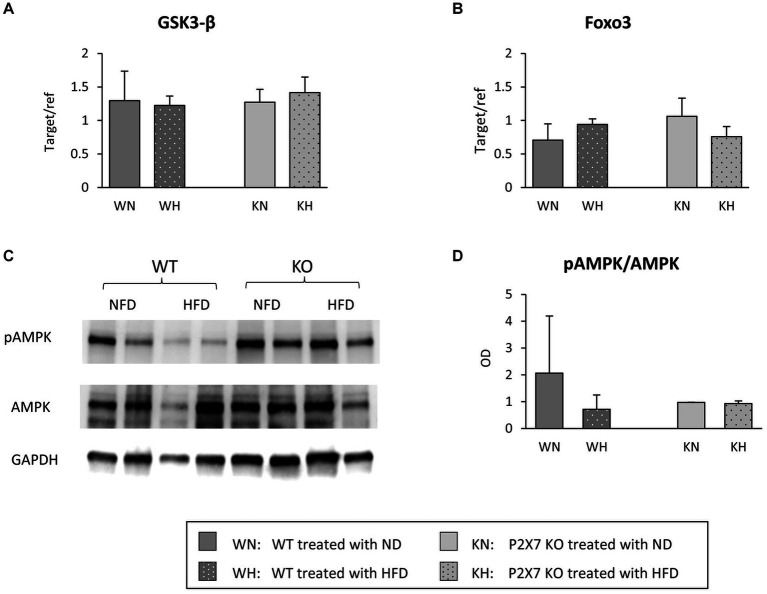
Substantia Nigra mRNA expression of GSK-3β **(A)** and Foxo3 **(B)** in WT and KO mice treated with normal (ND) or high-fat diet (HFD). Exemplificative Western blots of AMPK and its phosphorylated form are shown in **(C)**; the relative quantification of ratio pAMPK/AMPK is reported in **(D)**. Data are mean ± SD (*n* = 6 for each group). *p* = ns.

To give a mechanistic explanation of the altered morphology of TH neurons, we analyzed the principal downstream factors involved in the insulin/PI3/AKT pathway. Messenger of glycogen synthase kinase 3 (GSK3b) and fork head box (FOXO3) transcription factor, affecting a plethora of cellular functions downstream of insulin signal (23), in this brain area was not influenced by HFD (Figures 4A,B). On the other end, Western blot analysis of AMPK, a sensor molecule involved in maintaining energy balance (24), showed a downward trend in HFD-treated WT, while it was unchanged in KO mice (Figures 4C,D).

### Hippocampus

**Figure 5:**
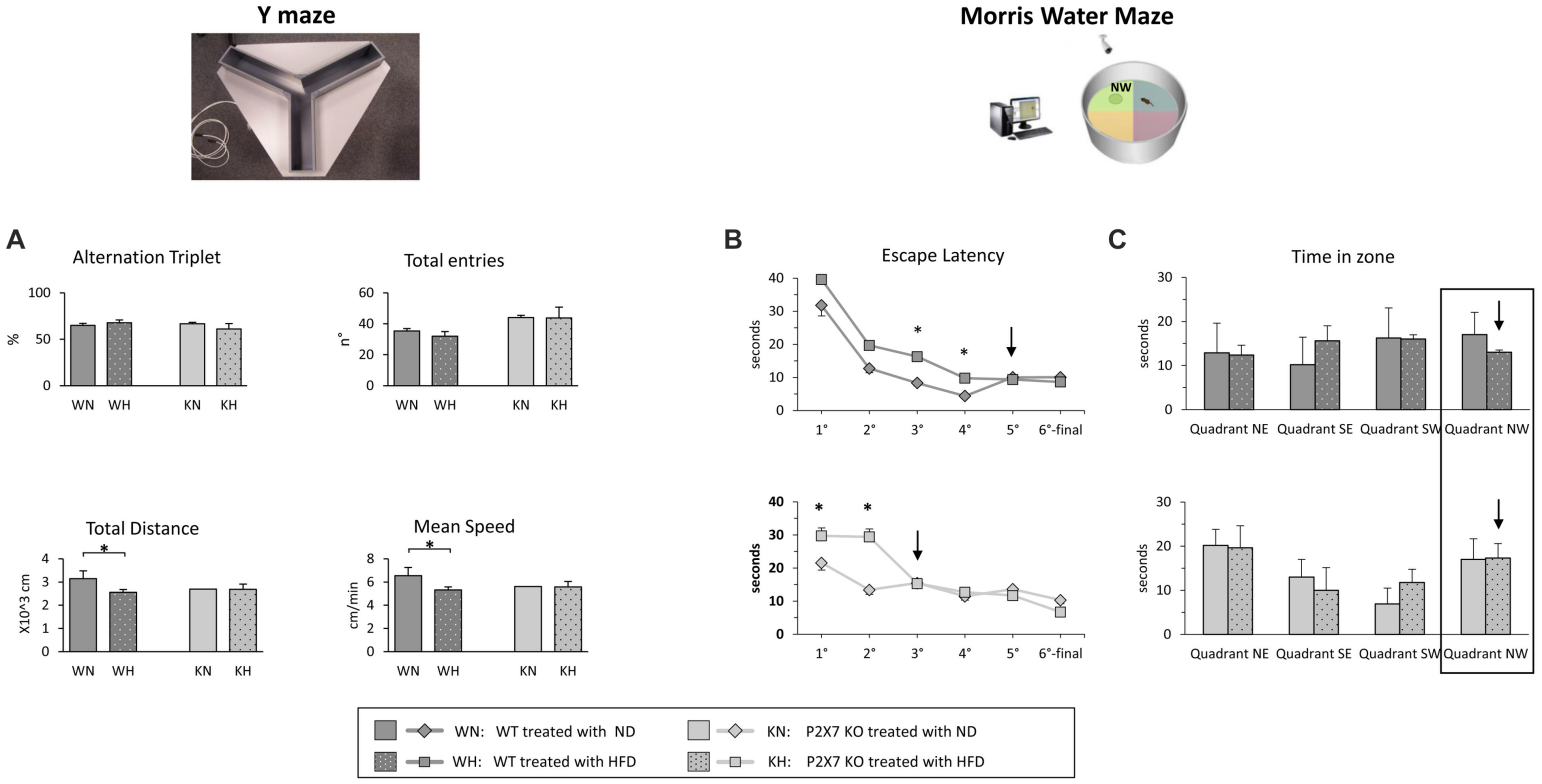
Y-Maze experiments **(A)**: percentage of correct alternation triplets and number of total entries (upper panels) and travelled total distance and mean speed (lower panels). Morris Water Maze experiments: the Escape Latency for each day of the trial (1st–5th) is shown in **B**; in the 6th final day, when the experiment was performed without platform, the two graphs report the time that animal required to cross the point where the platform was placed (Time in zone) **(C)**. Data are mean ± SD (*n* = 6 for each group). *p < 0.05.

At the end of dietary treatment, P2X7R KO and WT mice carried out two behavioral tests to evaluate their spatial working memory performance, linked to the hippocampal area. The Y-Maze test, a video tracking system that records animal’s movements in the maze and calculates some parameters (Figure 5A), was used. We did not observe significant differences between groups (WT vs. KO mice) or treatment (NFD vs. HFD) in the number of total entries in the Y-Maze arms and in the correct alternation triplets. Total distance travelled and mean speed resulted, instead, reduced in HFD-WT mice. In the Morris Water Maze test, the analyzed parameters were Escape Latency (the time required to find out the hidden platform in the pool) and Time in Zone (the time spent by each animal in the pool area where the platform was placed during training). The results are shown in Figures 5B,C. During the fifth day of training, WT animals fed with HFD reached the same performance of WT-NFD only the last day; conversely, P2X7R KO mice treated with HFD achieved the same performance of their NFD littermates, following 2 days of training (Figure 5B). In the last day, the experiment was performed without the platform in the pool, and the Time in Zone (in NW area) was recorded. P2X7R KO mice spent the same time in this area, independently of the treatment; conversely, WT animals fed with HFD spent less time in this quadrant with respect to their NFD-fed littermates (Figure 5C).

**Figure 6:**
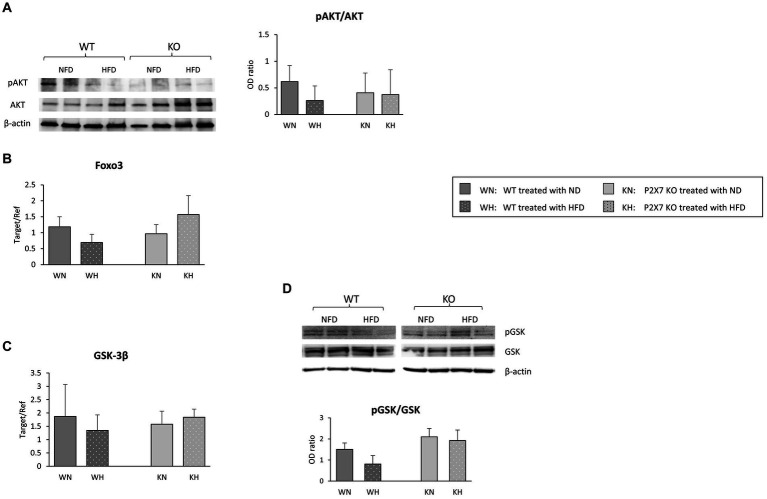
Western blot analysis of AKT/pAKT in the Hippocampus with relative quantification **(A)**. In the same area, mRNA expression of Foxo3 **(B)** and GSK-3β **(C)**; for the last molecule, protein quantification is also reported **(D)**. Data are mean ± SD (*n* = 6 for each group). *p* = ns.

We then repeated the analysis carried out in SN, looking at the proteins downstream to insulin pathway (PI3K/AKT). In fact, insulin regulates a variety of downstream signaling proteins that drive neuronal death and survival (23). In P2X7 KO mice, AKT signal did not vary after HFD, while WT mice showed a trend to a reduction in the pAKT/AKT ratio (Figure 6A); FOXO3, a transcription factor involved in cell survival, did not show any modification (Figure 6B).

In addition to a direct effect on glucose homeostasis, hippocampal GSK-3β activation has been associated with neurodegenerative diseases (25). In our experimental model, while HIP revealed the same GSK-3β mRNA level in each group of animals (Figure 6C), HFD seemed to induce a downward trend in the pGSK/GSK protein ratio in WT animals, while in KO mice, this trend was absent (Figure 6D).

## Discussion

The present study has addressed the role of the systemic P2X7R-inflammasome complex as mediator of some metabolic effects on the brain and, in particular, the connection between insulin signaling and low-grade inflammation. To this aim, WT and P2X7R KO mice were treated with normal chow and HFD, a stimulus able to induce an inflammatory damage at the level of various brain areas, e.g., the Substantia Nigra and Hippocampus. The study provides novel knowledge about the role played by P2X7R activation in the pathogenesis of neuronal disorders. We show in this study that a generic inflammatory stimulus, induced by a high content of fats in the diet, triggers specific modifications of insulin-IGF pathways in different brain areas of mice, with repercussion on both neuronal morphology and function; these modifications are not observed in mice lacking P2X7R.

In our experimental model, as already reported (26), high fat diet produced weight gain, increased cholesterol and impaired IPGTT, without significative differences between WT and P2X7R KO animals; the use of a P2X7R antagonist in WT animals treated with HFD did not modify such parameters, confirming no role for P2X7R in mediating these metabolic effects. However, KO animals showed a higher expression in the insulin-IGF pathways in the Hippocampus, a brain area related to the pathogenesis of Alzheimer’s disease, and this was true irrespective of diet, suggesting for these molecules a protective role toward functional alterations induced by HFD. Indeed, in post-mortem brain specimens of AD patients, Steen et al. reported a reduction of mRNA for insulin, Igf-1 and relative receptors in the Hippocampus and other brain areas (27). Conversely, in the Substantia Nigra, we observed a quite similar expression of these pathways in WT and KO animals, suggesting a less relevant role of such brain area in the regulation of insulin signal. This resonates with a previous report (28), describing a dysregulation in glucose metabolism with normal insulin levels in non-diabetic subjects affected by Parkinson disease. In the Substantia Nigra, Igf-1 resulted overexpressed after four months of HFD only in KO mice. An elevation of IGF-1, a peptide involved in growth, maturation and survival of neurons, has been shown in an early phase of Parkinson’s disease in humans, gradually decreasing in the middle/late stages of the disease (29); this could be regarded as a compensatory mechanism aimed at protecting dopaminergic neurons from initial degeneration.

Regarding the molecular pathways mediating damage in these specific brain areas, besides α-synuclein, we evaluated the effect of HFD on Parkin, whose genetic mutations have been related to familial and sporadic forms of Parkinson (30, 31). A relationship between P2X receptors and Parkin, with putatively different meaning, has also been described in cellular models: in PC12 cells, Parkin potentiates the activity of P2X receptors involved in neurotransmitter secretion (32); more recently, in SH-SY5Y cells, it has been reported that Parkin degradation in proteasome induced by P2X7R activation might induce a deregulation of mitophagy, resulting in neuronal cell death (33). In our P2X7R KO model, HFD induced a marked increase in Parkin expression at the level of Substantia Nigra. It might be hypothesized that the HFD-induced raise in Parkin expression, observed in P2X7R KO mice, might protect them toward the formation of α-synuclein aggregates, In fact, the α-synuclein amount does not differ in the Substantia Nigra of WT and P2X7R KO animals (Figure 3A); such shield is lacking in WT animals, in which P2X7R is present and functioning.

The involvement of P2X7R in the pathophysiology of Parkinson’s disease is documented. In a rat model of PD, the activation of P2X7R promotes death of nigrostriatal dopaminergic neurons, and its inhibition by Brilliant Blue G attenuates microglial activation and loss of dopaminergic neurons (34); another report shows as P2X7R antagonism can revert hemiparkinsonian behavior and increases the immunoreactivity for tyrosine hydroxylase in the Substantia Nigra (35). This observation emphasizes the importance of neuroinflammation in these processes. To confirm a role of P2X7R as pro-inflammatory mediator, we analyzed the expression of NLRP3 and IL-1β, main components of the inflammasome. Although IL-1β did not differ in the two strains, a trend of higher NRLP3 mRNA and P2X7R/NRLP3 immunoreactivity after HFD, although not statistically significative, was observed in WT animals, confirming that neuroinflammation participates in such damage. The lack of statistically significant differences we observed is likely due to our mild experimental conditions; indeed, Kao et al. reported a significant increase in the number of Iba-1 positive cells and a reduction of dopaminergic neurons, compromising cognitive function and behaviors after a much longer treatment with HFD (20 weeks), started immediately after weaning (36). Of note, microglial NRLP3 inflammasome activation is critical for dopaminergic neuronal loss in a murine model of Parkinson’s disease (37) and we have recently shown an increased mRNA and protein expression of the P2X7R-NLRP3 inflammasome in lymphomonocytes of treatment-naïve Parkinson’s disease subjects, suggesting that even extra-neural P2X7R might be relevant in the early phase of the disease (38).

In the hypothesis of a minor role of insulin in determining the alterations observed in the Substantia Nigra, factors downstream the activation of insulin receptor, like glycogen synthase kinase 3 (GSK3β) and fork head box (FOXO3) transcription factor were not influenced by HFD; on the other hand, a reduction in pAMPK/AMPK after HFD in WT mice was observed. AMPK, a key cellular energy sensor, may be relevant when a bioenergetics failure occurs (39), and pAMPK is selectively and significantly reduced in the midbrain of Parkin^−/−^ mice, and can be restored by oral treatment with metformin, an AMPK activator (24, 40); we might speculate that, in P2X7R KO mice, Substantia Nigra is protected by a high expression of Parkin (Figure 2B).

In human studies, it has been shown that HFD is associated with worse performance on a cognitive task and increased risk of dementia. The biological mechanisms underlying this cognitive impairment, explored in animal models, involve inflammation, oxidative stress, insulin resistance, and impaired vascularization (41). In Wistar rats, tyrosine hydroxylase reduction levels in the nigrostriatal axis can be attributed to brain inflammation and oxidative/nitrosative stress (42). In a mouse model of AD, metabolic disorders and insulin resistance induced by HFD play a detrimental role on the development of Aβ and Tau pathology and neuroinflammation (43). A secondary role in such global neuroprotection might be exerted by the macrovasculature, and we have observed, through ultrasonography, that P2X7 KO mice show a preserved macrovascular hemodynamics (Solini, personal unpublished data). Alteration in the Hippocampus participates in several neurodegenerative diseases. Overweight and insulin-resistant rats and HFD-treated mice show poorer performances than control animals in spatial learning ability task, with reduced hippocampal plasticity (16, 44) and impaired working memory and cognition, associated with a Hippocampus dysregulation and increased mRNA expression of various pro-inflammatory cytokines/mediators (17). In our model, behavioral tests showed for the first time that P2X7R deletion could protect the Hippocampus from the detrimental effects of HFD (Figure 5); probably, we did not record statistically significant differences for all parameters analyzed because animals started HFD post weaning, a life period less sensitive to detrimental effects of such diet (45).

Being neurons high energy-consuming cells, glucose homeostasis plays an important role in neural activity, and its dysregulation can interfere with learning and memory processes (46). Following the working hypothesis that a low-grade stress as HFD could also impact on insulin pathway, we might speculate that P2X7R KO mice did not display significant differences in behavioral test related to the Hippocampus because of a major insulin and insulin receptor gene expression in this brain area with respect to WT mice. Indeed, heterozygous mice for insulin receptor showed a poor performance in a behavioral recognition test (47).

The PI3/AKT pathway is associated with synaptic plasticity and long-term memory, and their dysregulation might lead to detrimental consequences to neuronal growth, survival, and plasticity (48). In P2X7 KO mice, AKT signal did not vary after HFD, while WT mice showed a reduction in the pAKT/AKT ratio, although not statistically significant; FOXO3, a transcription factor downstream insulin signal, did not vary.

In addition to a direct effect on glucose homeostasis, hippocampal GSK-3β activation has been associated with Tau phosphorylation (49) and plaque accumulation. P2X7R inhibition or deletion, by promoting a selective GSK-3β phosphorylation block, avoids plaque accumulation (25); a memory restoration has been also reported after GSK-3β inactivation (50). Our observation of a trend for a reduced GSK-3β phosphorylation in the Hippocampus of WT animals after HFD agrees with these reports.

In summary, we report for the first time that HFD induces damage in dopaminergic neurons of Substantia Nigra and a worse cognitive performance, both attenuated in P2X7R KO mice; involved mechanisms might differ in the two brain areas, with a predominant role of inflammation in Substantia Nigra and a metabolic derangement in Hippocampus. The major limitations of our study include the small number of animals and the small brain size of mice, precluding a more detailed analysis of all potentially involved pathways. Moreover, we would underline as our results are obtained in an animal model of diet-mediated neuroinflammation and require to be confirmed in specific murine models of degenerative diseases such as Alzheimer’s disease and Parkinson’s disease.

## Data availability statement

The raw data supporting the conclusions of this article will be made available by the authors, without undue reservation.

## Ethics statement

The animal study was approved by Italian Minister for Animal Care (authorization number 943/2015-PR). The study was conducted in accordance with the local legislation and institutional requirements.

## Author contributions

CR: Conceptualization, Supervision, Writing – original draft, Data curation, Investigation, Methodology. MD: Data curation, Investigation, Methodology, Writing – original draft. FR: Data curation, Investigation, Methodology, Formal analysis, Writing – original draft. CK: Formal analysis, Investigation, Methodology, Project administration, Resources, Writing – original draft. FF: Investigation, Methodology, Project administration, Resources, Data curation, Writing – original draft. AS: Project administration, Resources, Conceptualization, Funding acquisition, Supervision, Validation, Writing – original draft, Writing – review & editing.

## Figures and Tables

**Table 1:** 

AMPK	AMP-activated protein kinase
AUC	area under curve
AD	Alzheimer’s disease
AST	aspartate aminotransferase
BDNF	brain-derived neurotrophic factor
CNS	central nervous system
EL	escape latency
FOXO3	fork head box 3
GFAP	glial fibrillary acidic protein
GSK3β	glycogen synthase kinase 3
HFD	high fat diet
HIP	hippocampus
HRP	horseradish peroxidase
IL-1β	interleukin-1β
IPGTT	intraperitoneal glucose tolerance test
MWM	Morris water maze
NFD	normal fat diet
NRLP3	NLR family pyrin domain containing 3
OCT	optimal cutting temperature
PBS	phosphate buffer
PD	Parkinson’s disease
PVDF	polyvinyl difluoride
P2X7R	P2X7 receptor
RT	reverse transcripted
ROS	reactive oxygen species
SN	substantia nigra
TH	tyrosine hydroxylase
WB	Western blot
WT	wild type
