# Supplementary material for: Lacking P2X7-receptors protects substantia nigra dopaminergic neurons and hippocampal-related cognitive performance from the deleterious effects of high-fat diet exposure in adult male mice
Source: Front Nutr. 2024 Jan 25;11:1289750. doi: 10.3389/fnut.2024.1289750 (PMC10854005; doi:10.3389/fnut.2024.1289750)
Supplement: Supplementary file 1 [file Data_Sheet_1.PDF]

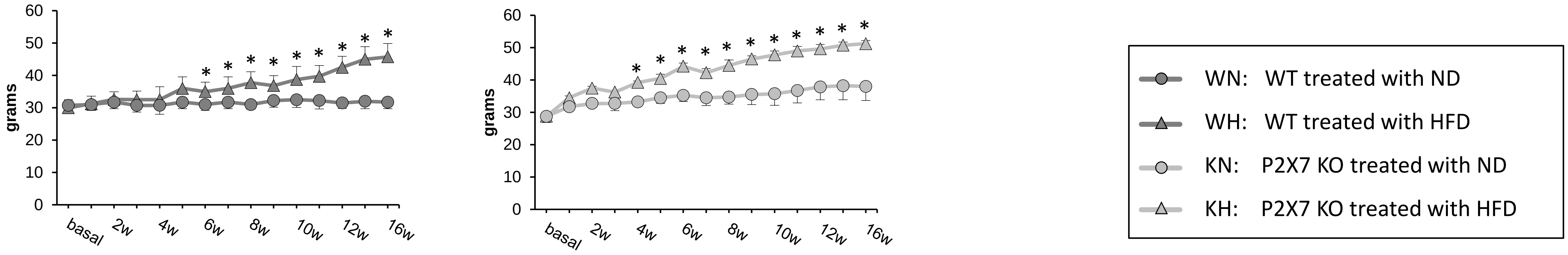

Suppl Figure 1 Body weight measured weekly during the dietary treatment in all experimental groups (n=6 for each group).

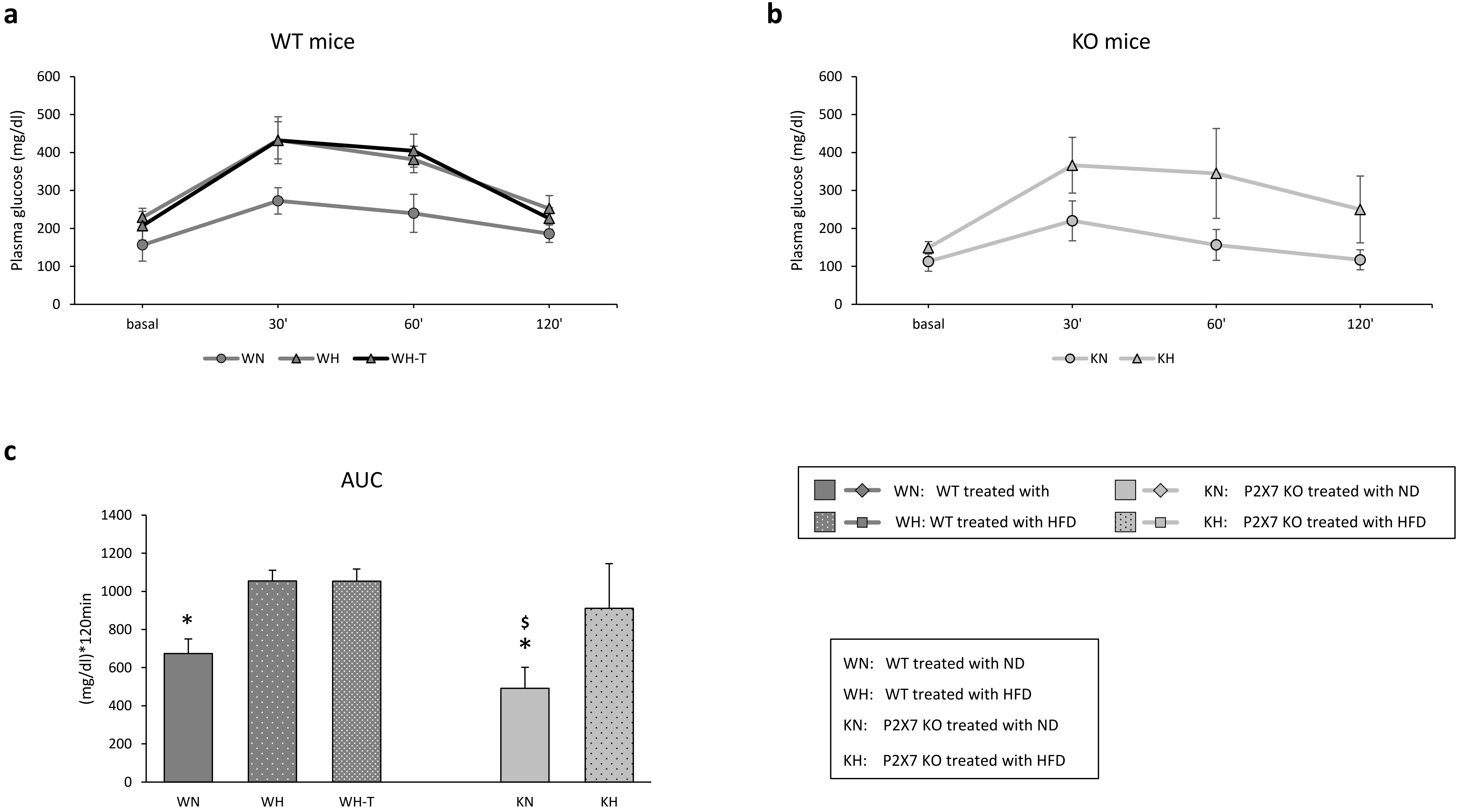

Suppl Figure 2 Intraperitoneal Glucose Tolerance Test (IPGTT) performed after four months of normal (ND) or high fat diet (HFD) in WT (**a**) and P2X7R KO (**b**) mice. WH-T indicates animals treated with high fat diet and P2X7 receptor antagonist. Quantification of area under curve (AUC) is reported in **c**. Data are mean±SD ((n=6 for each group, n=5 in the WH-T group). *p<0.01 normal *vs* high fat diet. $^{\$}$p<0.05 KN *vs* WN

Suppl Table 1 Parameters monitored during the dietary treatment at baseline and after four months in the four groups of mice (n=6 for each group).

	**WT BASAL**			**WT 4 MONTHS**			**KO BASAL**			**KO 4 MONTHS**		
	NFD	HFD	p	NFD	HFD	p	NFD	HFD	p	NFD	HFD	p
**Weight**	29 ± 1.9	28 ± 2.7	n.s.	34 ± 3.6	46 ± 4.1	0.011	29 ± 1.9	29 ± 0.8	n.s.	38 ± 4.2	51 ± 1.5	0.012
**Plasma Glucose**	158 ± 13	150 ± 10	n.s.	186 ± 70	204 ± 35	n.s.	197 ± 41	203 ± 30	n.s.	139 ± 21	194 ± 58	n.s.
**Triglycerides**	120 ± 24	135 ± 34	n.s	179 ± 44	158 ± 40	n.s	148 ± 9	163 ± 32	n.s.	188 ± 49	229± 43	n.s.
**Cholesterol**	155 ± 10	145 ± 8	n.s.	159 ± 17	178 ± 13	0.046	165 ± 20	178 ± 13	n.s.	157 ± 5	192 ± 23	0.012
**sGOT**	69 ± 20	88 ± 26	n.s.	97 ± 36	114 ± 58	n.s.	113 ± 38	86 ± 30	n.s.	107 ± 31	134 ± 53	n.s.
